# Stochastic modulation of oscillatory neural activity

**DOI:** 10.1186/1471-2202-15-S1-P80

**Published:** 2014-07-21

**Authors:** Jérémie Lefebvre, Axel Hutt, Kevin Whittingstall, Micah M Murray

**Affiliations:** 1 Laboratory for Investigative Neurophysiology, Centre Hospitalier Universitaire Vaudois, Lausanne, 1011, Switzerland; 2 INRIA CR Nancy - Grand Est, Team NEUROSYS, Villers-les-Nancy, 54600, France; 3 Department of Diagnostic Radiology, University of Sherbrooke, Sherbrooke, Québec, Canada

## 

Rhythmic neural activity plays a central role in neural computation. Oscillatory activity has been associated with myriad functions such as homeostasis, attention, and cognition [1] as well as neurological and psychiatric disorders, including Parkinson’s disease, schizophrenia, and depression [2]. Despite this pervasiveness, little is known about the dynamic mechanisms by which the frequency and power of ongoing cyclical neural activity can be modulated either externally (e.g. external stimulation) or via internally-driven modulatory drive of nearby neurons. While numerous studies have focused on neural rhythms and synchrony, it remains unresolved what mediates frequency transitions whereby the predominant power spectrum shifts from one frequency to another.

Here, we provide computational perspectives regarding responses of cortical networks to fast stochastic fluctuations (hereafter “noise”) at frequencies in the range of 10-500 Hz that are mimicked using Poisson shot-noise. Using a sparse and randomly connected network of neurons with time delay, we determine the functional impact of these fluctuations on network topology using mean-field approximations. We show how noise can be used to displace the equilibrium activity state of the population: the noise smoothly shifts the mean activity of the modeled neurons from a regime dominated by inhibition to a regime dominated by excitation. Moreover, we show that noise alone may support frequency transition via a non-nonlinear mechanism that operates in addition to resonance. Surprisingly, stochastic fluctuations non-monotonically modulate network’s oscillations, which are in the beta band. The system’s frequency is first slowed down and then accelerated as the stimulus intensity and/or rate increases. This non-linear effect is caused by combined input-induced linearization of the dynamics and enhanced network susceptibility.

Our results provide insights regarding a potentially significant mechanism at play in synchronous neural systems; ongoing activity rhythms can be externally and dynamically modulated, and moreover indicate a candidate mechanism supporting frequency transitions. By altering the oscillation frequency of the network, power can be displaced from one frequency band to another. As such, the action of noise on oscillating neural systems must be regarded as strongly non-linear; its action recruiting more than resonance alone to operate on ongoing dynamics.
